# Molekulare Mechanismen der Entstehung und Verbreitung von Antibiotikaresistenz

**DOI:** 10.1007/s00103-026-04231-9

**Published:** 2026-04-09

**Authors:** Anke Heisig, Peter Heisig

**Affiliations:** Pharmazeutische Biologie und Mikrobiologie, Institut für Biochemie und Molekularbiologie, Universität Hamburg, Bundesstraße 45, 20146 Hamburg, Deutschland

**Keywords:** Antibiotika, Resistenzmechanismen, Resistenzentwicklung, Bakteriophagen, Infektionserreger, Antibiotic, Resistance mechanisms, Resistance development, Bacteriophages, Bacterial pathogens

## Abstract

Antibiotika zeichnen sich durch ihre selektive Toxizität gegenüber bakteriellen Infektionserregern aus. Aufgrund der hohen genetischen Flexibilität von Bakterien, die sowohl Mutationen in eigenen Genen als auch die Aufnahme fremder Gene aus anderen Spezies umfasst, können sich die Erreger rasch anpassen, etwa durch Veränderungen molekularer Zielstrukturen. Dieser Artikel gibt einen Überblick über die molekularen Grundlagen wichtiger, klinisch relevanter Resistenzmechanismen gegen Antibiotika und beschreibt die Verbreitungswege der beteiligten Gene.

Die Entstehung antibiotikaresistenter Mutanten lässt sich durch 3 molekulare Grundmechanismen erklären: (1) Änderung der Bindungsstelle für ein Antibiotikum, (2) Reduktion der Antibiotikumkonzentration an der Bindungsstelle und (3) enzymatische Modifikation des Antibiotikums. Abhängig von der molekularen Struktur des Antibiotikums, seiner Wechselwirkung mit der Zielstruktur sowie der genetischen Ausstattung des jeweiligen Erregers existieren zahlreiche Varianten dieser Mechanismen.

Für die Auswahl einer geeigneten Therapie bei Vorliegen einer Antibiotikaresistenz sind sowohl die Kenntnis des zugrunde liegenden Resistenzmechanismus als auch eine zeitnahe Identifizierung des verantwortlichen Erregers und seiner Empfindlichkeit gegenüber alternativ einsetzbaren Antibiotika erforderlich. Ein grundlegender Nachteil chemisch definierter Antibiotika besteht darin, dass sie sich strukturell nicht an durch Resistenz veränderte Bedingungen anpassen können. Neben der Reduktion des Selektionsdrucks und der Einhaltung hygienischer Standards stellt der Einsatz von Bakteriophagen eine wichtige Alternative dar. Als „biologische Wirkstoffe“ können sie sich während der Therapie durch genetische Veränderungen an resistente Bakterien anpassen.

**Zusatzmaterial online:**

Zusätzliche Informationen sind in der Online-Version dieses Artikels (10.1007/s00103-026-04231-9) enthalten.

## Abstract (en)

Antibiotics are characterized by their selective toxicity against bacterial pathogens. Due to the high genetic flexibility of bacteria, which includes mutations in their own genes as well as the uptake of foreign genes from other species, pathogens can rapidly adapt, for example, by altering molecular target structures. This article provides an overview of the molecular basis of important, clinically relevant mechanisms of resistance to antibiotics and describes the ways for transfer of the genes involved.

The emergence of antibiotic-resistant mutants can be essentially explained by three fundamental molecular mechanisms: (1) alteration of the binding site for an antibiotic, (2) reduction of the antibiotic concentration at the binding site, and (3) enzymatic modification of the antibiotic. Depending on the molecular structure of the antibiotic, its interaction with the target structure, and the genetic makeup of the respective pathogen, numerous variations of these three mechanisms exist.

To select an appropriate therapy in the presence of antibiotic resistance, the knowledge of the underlying resistance mechanism, the timely identification of the responsible pathogen and its susceptibility to alternative antibiotics are necessary. A fundamental disadvantage of chemically defined antibiotics is their inability to structurally adapt to conditions altered by the acquisition of resistance. In addition to reducing selective pressure and maintaining hygienic standards, the use of bacteriophages represents a promising alternative. As “biological agents,” phages can adapt to phage-resistant bacterial mutants during therapy through genetic changes.

## Einleitung

Paul Ehrlichs „Zauberkugeln“ mit dem Wirkstoff Arsphenamin stellten im Jahr 1909 einen ersten Arzneistoff mit spezifischer Wirksamkeit gegen den Syphiliserreger *Treponema pallidum* dar [1]. Revolutionär war der Ansatz einer selektiven Therapie, die gezielt auf einen Mikroorganismus (z. B. Bakterien) in einem Makroorganismus (Mensch bzw. Tier) ausgerichtet ist. Aus Beobachtungen einer selektiven Anfärbbarkeit von Bakterien mit chemisch definierten Farbstoffen schloss Ehrlich auf eine selektive Wechselwirkung dieser Substanzen mit bakteriellen Strukturen – analog zur spezifischen Bindung anderer Arzneistoffe an Zielstrukturen in menschlichen Zellen. Die haploide genetische Information der Bakterienzellen kann jedoch mit relativ hoher Rate Veränderungen erwerben, die u. a. Antibiotikaresistenz vermitteln. Unter dem Selektionsdruck einer Antibiotikatherapie werden entsprechende Mutanten aus einer bakteriellen Population bevorzugt selektiert.

In diesem Artikel soll an ausgewählten Beispielen ein Überblick über die molekulargenetischen Grundlagen wichtiger, klinisch relevanter Resistenzmechanismen gegen Antibiotika und über Verbreitungswege der beteiligten Gene gegeben werden. Solche Kenntnisse ermöglichen nicht nur ein besseres Verständnis für Entstehung und Verbreitung von Multiresistenz, sondern schaffen auch die Grundlagen für das Design Resistenz-refraktärer neuer oder modifizierter Wirkstoffe.

## Resistenz gegen Antibiotika – eine Frage der Definition

Der Begriff „Antibiotikaresistenz“ bezeichnet allgemein die relative Unempfindlichkeit von Bakterienzellen für ein Antibiotikum im Vergleich zu anderen Zellen mit höherer Empfindlichkeit für das gleiche Antibiotikum. Quantitativ wird die jeweilige Empfindlichkeit von Bakterien als die geringste Konzentration eines Antibiotikums angegeben, die eine Wachstumshemmung bewirkt. Diese wird als *minimale Hemmkonzentration* (MHK in µg/ml) bezeichnet.

Für die therapeutische Anwendung von Antibiotika wird zwischen *klinischer Empfindlichkeit* und *klinischer Resistenz* unterschieden. Als Bezugsgröße dient die bei vorgegebener Dosierung am Infektionsort erreichbare Konzentration eines Antibiotikums. Liegt die MHK unter dieser Konzentration, wird eine Zunahme der Zellzahl durch Teilung der Einzelzellen gehemmt (bakteriostatischer Wirkungstyp) oder reduziert (bakterizider Wirkungstyp), was als *klinische Empfindlichkeit* bezeichnet wird. Liegt die MHK darüber, können sich die Erreger weiterhin teilen (*klinische Resistenz*). Betrifft klinische Resistenz alle Stämme einer Spezies, liegt eine *natürliche* oder *primäre Resistenz *vor. Verantwortlich dafür sind Spezies-spezifische genetische Eigenschaften, z. B. Variationen im Gen für eine Zielstruktur. Finden sich Mutationen nur bei dem aktuell infizierenden Stamm einer Spezies, liegt eine *sekundäre*, d. h. *erworbene* Resistenz gegenüber einem Antibiotikum vor.

Die Bestimmung der MHK eines Antibiotikums für ein Bakterienisolat erfolgt nach einer standardisierten direkten Methode als Bouillon-Dilutionstest. Dazu wird von einer Stammlösung eines Antibiotikums durch serielle 1:2-Verdünnung in definierter Nährbouillon eine Konzentrationsreihe erstellt und mit einer vorgegebenen Zellzahl pro ml (*Inokulum*) des Teststammes beimpft. Nach Inkubation unter vorgegebenen Bedingungen (Zeit, Temperatur) wird die MHK als geringste Konzentration des Antibiotikums ermittelt, die eine Hemmung des Bakterienwachstums, erkennbar als fehlende Trübung, bewirkt. Bei ausbleibender Trübung kann zunächst nicht zwischen *bakteriostatischer *und *bakterizider Wirkung *unterschieden werden. Dazu ist anschließend zu prüfen, ob aus dem ungetrübten Ansatz auf Nährmedium ausgeimpfte Proben Wachstum von Bakterienzellen zeigen oder nicht.

Die experimentellen Bedingungen der MHK-Bestimmung mittels Bouillondilution sind aus Gründen der Vergleichbarkeit zwischen verschiedenen Laboratorien genormt, z. B. nach Vorgaben des European Committee on Antimicrobial Susceptibility Testing (EUCAST; [2]) bzw. des Clinical & Laboratory Standards Institute (CLSI; [3]). Für bestimmte Erreger, wie *Mycobacterium tuberculosis *werden jedoch wegen des sehr langsamen Wachstums alternativ DNA-Sequenzen bekannter Resistenz-assoziierter Gene aus klinischen Proben bestimmt [4]. Auf diese Weise kann zeitnah der Resistenzgenotyp ermittelt werden. Anhand bekannter Korrelationen zwischen spezifischen Mutationen und der damit assoziierten MHK lässt sich daraus auf den Resistenzphänotyp schließen. Dies ermöglicht den frühzeitigen Beginn einer kalkulierten Therapie.

## Resistenz – Besonderheit einer Therapie mit Antibiotika

Bei Anwendung eines Antibiotikums in einer Konzentration oberhalb der MHK tragen vor allem 2 Faktoren dazu bei, dass nach Teilung einer „Mutterzelle“ einzelne „Tochterzellen“ nach Erwerb von Mutation(en) wachsen (Resistenz):

1. **Direkte Ausprägung des Phänotyps einer genotypischen Veränderung.**
  Bei den haploiden Bakterienzellen führt die Veränderung einer einzelnen Base eines DNA-Strangs dazu, dass bei der nachfolgenden DNA-Replikation 2 unterschiedliche Tochterstränge vorliegen. Nach Abschluss der Replikation wird jeweils eines dieser doppelsträngigen (ds) DNA-Moleküle in eine Tochterzelle verteilt, von denen eine die ursprüngliche DNA-Sequenz besitzt (*Wildtyp*), während die andere die Mutation trägt (*Mutante*). Durchschnittlich findet sich in einer Bakterienkultur eine solche mutierte Zelle in 10^7^–10^10^ Zellen [5].
2. **Hohe Wahrscheinlichkeit für zufällige Entstehung resistenter Mutanten in großen Populationen**.
  Aufgrund einer im Vergleich zu humanen Zellen sehr hohen Verdopplungsgeschwindigkeit fast aller bakteriellen Erreger (2–3 Teilungen/h) werden innerhalb weniger Stunden sehr hohe Zellzahlen (≥ 10^9^ Zellen/ml) erreicht. Dadurch besteht eine ausreichend große Wahrscheinlichkeit, dass in einer solchen Zellpopulation spontan und ungerichtet Mutanten entstehen, die gegen ein Antibiotikum resistent sind und im Verlauf der Therapie durch dieses selektiert werden.

Bei länger andauernder bzw. wiederholter Selektion durch ein Antibiotikum *in vitro* werden aus großen Zellpopulationen resistente Mutanten selektiert. *In vivo* bieten eine stabile Mikroflora am Infektionsort sowie ein aktives Immunsystem Schutz vor der Ausbreitung resistenter Mutanten. Unnötiger Selektionsdruck durch Antibiotikaanwendung bei nicht bakteriell bedingten Infektionen oder zu nichttherapeutischen Zwecken (z. B. Tiermast) muss deshalb vermieden werden, um den Eintrag von Antibiotika sowie Antibiotikaresistenzgenen in die Umwelt über Ausscheidungen und Abwasser zu reduzieren.

## Übertragbarkeit von Antibiotikaresistenzgenen

Neben chromosomaler DNA tragen Bakterien häufig extrachromosomale DNA in Form von Plasmiden, zwischen denen innerhalb einer Zelle Antibiotikaresistenzgene übertragen werden können. Auch zwischen Bakterienzellen einer Generation können Resistenzgene horizontal über 3 Wege übertragen werden: 1) Transformation, 2) Konjugation und 3) Transduktion.

### Transformation.

Durch Transformation gelangt freie DNA, z. B. als chromosomales Bruchstück aus abgestorbenen Bakterienzellen (Donor) über spezielle Aufnahmesysteme in eine andere Zelle (Rezipient) der gleichen oder einer verwandten Art (z. B. von Penicillin-resistenten *Streptococcus pneumoniae *auf Penicillin-sensible *Streptococcus-*Arten der oralen Mikroflora; [6]). Dabei wird das Gen für ein empfindliches Penicillin-Bindeprotein im Chromosom der Empfängerzelle über homologe Rekombination gegen das aufgenommene resistente Allel aus dem Genom des Donors ausgetauscht, die Rezipientenzelle wird Penicillin-resistent.

Freie DNA kann auch als ringförmige Plasmid-DNA aufgenommen werden und sich extrachromosomal replizieren. Während einer Replikationsrunde werden pro Chromosom Plasmide größenabhängig tausendfach (sehr kleine Plasmide, < 1000 Basenpaare (bp), „multi-copy“), mindestens aber einmal (sehr große Plasmide, > 1.000.000 bp, „single copy“) repliziert. Liegt ein Gen für Antibiotikumresistenz auf einem Multi-copy-Plasmid, werden alle Genkopien in einer Zelle gleichzeitig exprimiert, was hohe Resistenz bewirkt.

Für die Mobilisierung von Antibiotikaresistenzgenen innerhalb einer Zelle wie auch zwischen Zellen spielen mobile genetische Elemente (Integrons, Transposons) eine Rolle. Kurze repetitive DNA-Sequenzen flankieren jeweils ein Resistenzgen. Durch Rekombination zwischen solchen Sequenzen unterschiedlicher Gene können diese zu mobilen Multiresistenz-Genkassetten akkumulieren, die mittels Plasmid-codierter Enzyme (Transposasen, Integrasen) auf andere DNA-Moleküle derselben oder einer anderen Zelle transferiert werden können [7]. Wird durch ein Antibiotikum Selektionsdruck erzeugt, können direkt benachbarte Gene auch ohne Selektionsdruck ebenfalls exprimiert werden, und eine Multiresistenz entsteht [8].

### Konjugation.

Die häufigste Form des horizontalen Gentransfers ist die Konjugation. Sie bezeichnet die direkte Übertragung zwischen 2 Zellen von (1) konjugativen Plasmiden oder von (2) „integrative conjugative elements“ (ICE), die im Chromosom integriert vorliegen, mit diesem mitrepliziert werden, jedoch unter bestimmten Umgebungsbedingungen enzymatisch aus dem Chromosom ausgeschnitten und zu konjugativen Plasmiden zirkularisiert werden. Bei gramnegativen Zellen wird für die Konjugation ein Plasmid-codierter kontraktiler Fertilitäts-(F-)Pilus auf der Oberfläche der Donorzelle verankert, der über Kontakt zu einer Rezipientenzelle beide Zellen zusammenbringt. Nach Spaltung eines Einzelstrangs des Plasmids in der Donorzelle wird dieser abgerollt („rolling circle“) und entlang des F‑Pilus in die Rezipientenzelle transportiert. ICEs werden zunächst rekombinativ aus dem Chromosom ausgeschnitten, zirkularisiert und dann wie ein konjugatives Plasmid in die Rezipientenzelle übertragen. Grampositive Zellen lagern sich direkt aneinander und transferieren den DNA-Einzelstrang unter Beteiligung eines Membran-assoziierten Typ-IV-Sekretionssystems [9]. Der lineare DNA-Einzelstrang in der Rezipientenzelle wird rezirkularisiert und wie der verbliebene, intakte Einzelstrang in der Donorzelle zu einem Doppelstrang vervollständigt. Plasmide verbleiben extrachromosomal (replikative Transkonjugation unter Rolling-circle-Replikation [10]), ICE-Moleküle werden wieder in das Chromosom integriert. So können mehrere Antibiotikaresistenzgene gleichzeitig zwischen Zellen auch verschiedener Spezies übertragen werden.

### Transduktion.

Ein rekombinativer DNA-Transfer unter Beteiligung von Bakteriophagen (Viren, die ausschließlich Bakterien infizieren; [11]) wird als Transduktion bezeichnet (siehe auch Artikel zu Bakteriophagen von P. Heisig und A. Heisig in diesem Themenheft). Bei der Transduktion können auch kurze DNA-Sequenzen des bakteriellen Chromosoms, wie z. B. Antibiotikaresistenzgene, zusammen mit der Phagen-DNA von der Donorzelle auf die Rezipientenzelle übertragen werden. Wenn ausreichende Homologie zur chromosomalen DNA vorhanden ist, können diese Sequenzen rekombinativ in das Chromosom eingekreuzt werden. Auf diese Weise können auch chromosomale Antibiotikaresistenzgene zwischen Zellen übertragen werden, selbst wenn die fehlverpackte Fremd-DNA essenzielle Phagengene ersetzt (Pseudophagen). Für eine stabile Etablierung ist es notwendig, dass eingekreuzte DNA in ein replizierbares Molekül wie das Chromosom oder ein Plasmid integriert wird. Verschiedene weitere Faktoren können die Ausbreitung von Antibiotikaresistenzeigenschaften – negativ oder positiv – beeinflussen (Tab. 1).

**Tab. 1:** Faktoren, die die Verbreitung von Antibiotikaresistenz beeinflussen

Faktoren	Beschreibung
Expression von Resistenzgenen – z. B. für Antibiotika-abbauende Enzyme, wie β‑Lactamasen	Wird ein Plasmid mit Resistenzgen von einer Donor- auf eine Rezipientenzelle übertragen, müssen regulatorische Nukleotid-Sequenzen für Transkription und Translation des Resistenzgens sowie für die Replikation der Plasmid-DNA funktionell aktiv sein. Bakteriophagen, wie T7, codieren für eine eigene RNA-Polymerase die nur T7-spezifische Gene exprimiert [40]
Inkompatibilität eines Plasmids im Rezipienten mit einem vom Donor aufgenommenen	Trägt eine Rezipientenzelle Plasmide einer Inkompatibilitätsklasse, kann ein zweites Plasmid der gleichen Inkompatibilitätsklasse nicht gleichzeitig etabliert werden. RNA-basierte Kontrollsysteme verhindern eine gleichmäßige, aktive Verteilung beider Plasmide auf beide Tochterzellen oder beide Plasmidtypen werden bei der Replikation ungleichmäßig auf die Tochterzellen verteilt. In beiden Fällen entstehen nachfolgend Tochterzellen mit nur einem Plasmidtyp
Inaktivierung übertragener Fremd-DNA	Primitive, über DNA-Restriktion/Modifikation [41] wirkende „Immunsysteme“ in Rezipientenzellen verhindern durch enzymatischen Abbau der neu aufgenommenen DNA die Koexistenz beider Plasmide
Antibiotikawirkung auf natürliche Mikroflora am Infektionsort	Die Konzentration eines therapeutisch eingesetzten Antibiotikums am Infektionsort beeinflusst die Zusammensetzung des dort vorhandenen, natürlich empfindlichen Anteils des Mikrobioms [42]. Dadurch kann die Ausbreitung resistenter Erregerzellen begünstigt sein, z. B. über eine Freigabe biologischer Nischen
Standortspezifische Bedingungen am Infektionsort oder Milieuveränderungen	Das Wachstumsverhalten infizierender Bakterien am Infektionsort unterliegt dem Einfluss verschiedener Parameter: Nährstoffangebot, jeweiliger Zellstoffwechseltyp, O_2_-Gehalt, pH-Wert und Ionenkonzentrationen können einen unterschiedlichen Einfluss auf die jeweiligen Erregerzellen haben. So bringt nicht jede genetische Veränderung (Mutation bzw. DNA-Aufnahme), die zu einer erhöhten Antibiotikaresistenz im In-vitro-Test führt, zugleich auch einen Selektionsvorteil am Infektionsort mit sich
Nebeneffekte von Resistenz-assoziierten Mutationen	Eine Mutation der Zielstruktur an der Bindestelle eines Antibiotikums kann zur Ausbildung einer weniger aktiven Proteinstruktur führen. Nimmt dadurch deren biologische Aktivität ab, kann dadurch die „Fitness“ der resistenten Mutante reduziert sein [43]
Nebeneffekte durch Aufnahme Plasmid-codierter Resistenzgene	Nach Aufnahme von Multi-copy-Plasmiden mit Resistenzgenen kann der zusätzliche Energiebedarf für deren Replikation zu verringerter Geschwindigkeit der Zellteilung führen. Ohne Selektionsdruck überwachsen schneller teilende Plasmid-freie Zellen die Plasmid-tragenden, resistenten, aber langsamer teilenden Zellen („Fitnessverlust“; [44])

### Identität und Resistenzphänotyp eines Erregers

Ein wesentlicher Faktor für die Stabilisierung des Resistenzphänotyps eines bakteriellen Erregers ist der vom Antibiotikum ausgeübte Selektionsdruck, der von mehreren Parametern bestimmt wird, wie:

1. Antibiotikakonzentration,
2. Wirkdauer eines Antibiotikums am Infektionsort,
3. Wirkungsspektrum und
4. Wirktyp des Antibiotikums.

So ist für Patienten mit eingeschränkter Immunabwehr der zeitnahe Beginn einer wirksamen Antibiotikatherapie von entscheidender Bedeutung. In der Regel sind jedoch der Erreger und seine Antibiotikaempfindlichkeit zu diesem Zeitpunkt noch nicht bekannt, weshalb zunächst eine kalkulierte Therapie mit einem Breitspektrum-Antibiotikum begonnen wird.

Nach Identifizierung des Erregers und Bestimmung seiner Antibiotikaempfindlichkeit ist es möglich, auf eine gezielte Therapie umzustellen, was die Erfolgsaussichten einer antiinfektiven Therapie deutlich erhöht [12]. Molekulargenetische Techniken, wie Next-Generation Sequencing (NGS) bzw. Multi-Locus Sequence Typing (MLST), wie sie z. B. für die Diagnostik von *Mycobacterium tuberculosis* eingesetzt werden (s. oben), ermöglichen einen schnellen Nachweis. Jedoch birgt eine punktuelle DNA-Sequenzanalyse bekannter Resistenz-assoziierter Genbereiche das Risiko, dass bislang nicht bekannte Mutationen nicht erfasst werden bzw. ihre Bedeutung für eine Resistenz nicht abgeschätzt werden kann. Dies gilt auch für neue Kombinationen bekannter Mutationen, die sowohl additive, synergistische oder antagonistische Effekte zeigen können.

## Drei Mechanismen der Antibiotikaresistenz mit molekularen Varianten

Die oben genannten genetischen Veränderungen – Mutationen sowie horizontaler Transfer von Resistenzgenen innerhalb einer Zelle, wie auch die o. a. 3 Übertragungswege zwischen Zellen – können jeweils einen von 3 Typen grundlegender Mechanismen bewirken, die zur Entstehung resistenter Bakterien führen:

1. Veränderung der Zielstruktur für das Antibiotikum,
2. Inaktivierung des Antibiotikums,
3. Einschränkung des Zugangs des Antibiotikums zur Zielstruktur.

Alle 3 Mechanismen (Typ 1, 2 und 3) weisen viele molekulare Varianten auf, die meist für bestimmte Antibiotika charakteristisch sind.

Für die Veränderung der Zielstruktur (Typ 1) genügen oft einzelne oder wenige Mutationen, die über strukturelle Veränderung die Bindungsaffinität für ein Antibiotikum reduzieren. Es gibt aber auch enzymatische Mechanismen und die Aufnahme und Expression neuer Gene. Die natürliche Aktivität/Funktion der Zielstruktur bleibt dabei erhalten.

### Typ 1: Veränderung der Zielstruktur für das Antibiotikum

Ein Resistenzmechanismus gegen das Aminoglycosid-Antibiotikum Streptomycin beruht auf einer Mutation im Gen für das RpsL-Protein der ribosomalen 30S-Untereinheit. Die veränderte Proteinstruktur von RpsL reduziert die Affinität des Antibiotikums zur Bindungsstelle. Die MHK von Streptomycin steigt um mehr als das 300-Fache [13].

Resistenz gegen das Polymyxinderivat Colistin wird durch eine enzymatische Modifikation der Zielstruktur Lipopolysaccharid (LPS) auf der Außenseite der äußeren Membran erreicht: Die natürliche, negative Oberflächenladung von LPS wird durch Kopplung mit positiv geladenen Molekülen (z. B. Phosphoethanolamin durch das Mcr-1-Enzym) deutlich reduziert bzw. in eine positive Netto-Ladung überführt, was die Bindung positiv geladener Colistinmoleküle verhindert. Die Einlagerung in die LPS-Schicht mit anschließender Desintegration kann nicht mehr erfolgen. Die Gene für Mcr-1-Typ-Enzyme sind überwiegend Plasmid-codiert und zwischen verschiedenen Bakterienarten transferierbar. Ein alternativer Mechanismus einer Colistinresistenz durch Änderung der Zielstruktur beruht auf quantitativer Veränderung: Eine K.-o.-Mutation im LPS-Biosynthese-Gencluster stoppt die LPS-Biosynthese an einem Zwischenschritt. In die strukturell veränderte äußere LPS-Schicht kann sich Colistin nicht einlagern [14].

Ursache für die bei *Staphylococcus aureus* weitverbreitete Resistenz gegen Penicillinase-feste Penicilline, wie z. B. Flucloxacillin, ist ebenfalls eine Veränderung der Zielstruktur durch Erwerb eines Transposons mit dem Gen für eine Flucloxacillin-unempfindliche Transpeptidase/Transglycosidase (Penicillin-Binde-Protein, PBP2a). Dabei ist neben diesem Enzym auch noch das *S.-aureus*-eigene, Flucloxacillin-empfindliche PBP2 aktiv und wird von dem Antibiotikum gehemmt. Das resistente PBP2a übernimmt dessen Funktion und sichert so das Überleben der Zellen („Dominanz“ des resistenten PBP2a-Allels; [15]).

Wichtigster Resistenzmechanismus in gramnegativen Bakterien gegen Fluorchinolone, wie Ciprofloxacin, sind eine Mutation in der primären Zielstruktur DNA-Gyrase („low-level resistance“) sowie eine zusätzliche Mutation in der sekundären, weniger empfindlichen Topoisomerase IV zusammen mit einer weiteren Gyrase-Mutation („high-level resistance“). Beide Zielstrukturen sind Typ-II-Topoisomerasen und bestehen aus 2 Paaren von Untereinheiten GyrA und GyrB bei Gyrase bzw. Homologen ParC und ParE (Topoisomerase IV). Das Holoenzym liegt in Form eines Tetramers A_2_B_2_ (bzw. C_2_E_2_) vor. Die wichtigste Resistenzmutation ist der Austausch der Aminosäure Serin gegen Leucin an Position 83 in der A‑Untereinheit (Nummerierung von *E. coli*). Anders als im Fall des PBP2a bei *Staphylococcus aureus* lässt sich Fluorchinolonresistenz nicht durch Übertragung des resistenten Allels von *gyrA*^R^ für eine Fluorchinolon-resistente Gyrase auf Zellen mit einer Fluorchinolon-empfindlichen Gyrase-A-Untereinheit (*gyrA*^*S*^) übertragen. In dieser Konstellation bleibt die Zelle Fluorchinolon-empfindlich, da die empfindliche Gyrase (GyrA^S^-Untereinheit) durch ein Fluorchinolon in einem Reaktionszwischenschritt kovalent an die DNA gebunden bleibt (bakterizide Wirkung), auch wenn das resistente Enzym die enzymatische Reaktion an anderen Stellen des Chromosoms aufrechterhält. Ausschlaggebend ist die dauerhafte Blockade der DNA-Replikation an der Bruchstelle mit Auslösen der „SOS-Stressreaktion“, die zum Zelltod führt („Dominanz“ des empfindlichen *gyrA*^*S*^-Gens; [16]). Dieselbe Reaktion zeigt auch eine Mutation der B‑Untereinheit der Gyrase und kann zu diagnostischen Zwecken genutzt werden [17].

Die Zielstruktur für das Makrolid-Antibiotikum Erythromycin kann dadurch „verändert“ werden, dass sie von Faktoren abgeschirmt wird, welche das Antibiotikum binden. So verändern ribosomale Schutzproteine vom ABC-F-Typ, wie MsrE, nach Bindung an Adenosintriphosphat (ATP) die Konformation der Makrolidbindestelle am Peptidyltransferase Center (PTC) der ribosomalen 50S-Untereinheit, sodass das Makrolid nicht mehr binden kann [18].

Ribosomale Schutzproteine („ribosomal protection proteins“, RPP), wie TetM und TetO, bewirken Tetracyclinresistenz durch Bindung ihres hochkonservierten C‑Terminus direkt an ein Nukleotid in der Tetracyclin-Bindestelle der 16S rRNA der ribosomalen 30S-Untereinheit und verdrängen dadurch gebundene Tetracyclinmoleküle [19].

„**Q**ui**n**olone-**r**esistance“-(Qnr‑)Proteine, als „Pentapeptid repeat protein“ bezeichnet, nehmen eine Struktur mit Homologie zu einem DNA-Doppelstrang an und stellen für Gyrase einen alternativen Bindungspartner dar, der jedoch nicht enzymatisch gespalten wird. Dadurch werden Gyrasemoleküle mit hoher Affinität im Komplex mit Qnr-Proteinen gebunden, weniger Gyrasemoleküle spalten und binden an freie DNA-Enden, was die MHK von Fluorchinolonen erhöht [20].

### Typ 2: Inaktivierung des Antibiotikums

Molekulare Mechanismen der Inaktivierung von Antibiotika (Typ 2) beruhen dagegen auf spezifischer enzymatischer Aktivität, wie z. B. β‑Lactamasen (Bla). Die häufigsten Typen sind Serin-Proteasen, von denen 3 Klassen A, C oder D mit einem hohen Grad an genetischer Variationsbreite existieren, sowie spezielle Zn^2+^-Proteasen der Klasse B mit breitem Wirkungsspektrum und Unempfindlichkeit für die meisten β‑Lactamase-Inhibitoren. Die Enzyme inaktivieren β‑Lactam-Antibiotika durch hydrolytische Spaltung des β‑Lactamrings. Diese Reaktion entspricht formal einer Transpeptidase-Reaktion von PBPs, jedoch wird das β‑Lactamase-Enzym durch hydrolytische Spaltung der transient gebildeten Esterbindung des Penicillin-β-Lactamase-Komplexes für weitere Reaktionen schnell wieder freigesetzt [21].

Neben solchen hydrolytischen Reaktionen zur Inaktivierung von Antibiotika findet sich eine Reihe von modifizierenden Reaktionen, bei denen funktionelle Gruppen (HO-, NH_2_− etc.) zu einer Inaktivierung von Antibiotika führen. Ein Beispiel sind Chloramphenicol-Acetyltransferasen (CAT-Enzyme), welche Chloramphenicol durch O‑Acetylierung zweier OH-Gruppen inaktivieren [22].

Ursachen für Makrolidresistenz wie Erythromycin (14-gliedriger Lactonring), Azithromycin (15-gliedrig) oder Tylosin (16-gliedrig) finden sich als Variationen aller 3 grundlegenden Mechanismen. Primär resistent sind die meisten gramnegativen Erreger aufgrund der geringen Permeabilität der äußeren Membran für die relativ großen, hydrophilen Makrolidmoleküle (eine Ausnahme bildet die Empfindlichkeit von *Haemophilus influenzae* für Azithromycin; [23]). Weiter verbreitet und klinisch wichtigster Resistenzmechanismus ist die enzymatische Modifikation der Makrolid-Zielstruktur durch *erm*-Genprodukte (**E**rythromycin**r**esistenz-**M**ethyltransferasen), die 2 Methylgruppen auf einen Adeninrest (A2058) der 23S-rRNA übertragen. Adenin-2058 bildet mit Nukleotiden in den Domänen II und V der 23S-rRNA (u. a. A2059, C2611, A752) sowie 2 Proteinen (L4, L22) der ribosomalen 50S-Untereinheit eine spezifische Bindungstasche für Erythromycin. Die Erm-katalysierte Methylierung von A2058 alleine bewirkt bereits klinisch relevante Resistenz vom MLS_B_-Typ, die neben **M**akroliden auch **L**incosamide (Clindamycin) und **S**treptogramin-**B-**(Quinupristin‑)Antibiotika umfasst [24]. MLS_B_-basierte Resistenz zeigt eine durch Makrolid induzierbare Expression einiger *erm*-Gene. Dieser als Attenuierung bezeichnete Induktionsmechanismus basiert darauf, dass die Translation eines zweiten, *erm* vorangestellten Gens in Anwesenheit von Makroliden vorzeitig gestoppt wird. Dadurch wird die ribosomale Bindungsstelle für die Expression des nachfolgenden *erm*-Gens aktiviert [25]. Weitere Veränderungen in der Bindungstasche können in seltenen Fällen auch durch Mutationen an den genannten Nukleotiden der 23S-rRNA zu Makrolidresistenz führen. Aufgrund der bei den meisten Erregern mehrfach vorhandenen Genkopien für die 23S-rRNA ist der Effekt jedoch gering und daher nicht von klinischer Bedeutung [24].

Ein anderer Resistenzmechanismus enzymatischer Inaktivierung von 14-gliedrigen Makroliden, wie Erythromycin sowie von Azithromycin, jedoch nicht von Ketoliden, besteht in der Spaltung der Esterbindung durch **E**rythromycin**r**esistenz-**E**sterasen (Ere-Enzyme), z. B. Ere(A) und Ere(C) [26].

Hauptmechanismus einer Resistenz gegen Aminoglycosid-Aminocyclitol-(AGAC-)Antibiotika, wie Gentamicin oder Amikacin, sind Aminoglycosid-modifizierende Enzyme (AME). Diese werden in AGAC-Phosphotransferasen (APH), -Acetyltransferasen (AAC) und -Nukleotidyltransferasen (ANT) unterteilt, welche OH- bzw. NH_2_-Gruppen verschiedener AGAC-Antibiotika modifizieren, die dadurch ihre antibiotische Aktivität verlieren. Das Enzym Aminoglycosid-N6′-Acetyltransferase Subtyp Ib (AAC6′-Ib) ist ein Beispiel dafür. Im Falle von AAC6′-Ib sind z. B. Kanamycin und Amikacin Substrate [27]. Durch nur 2 Punktmutationen wird das Enzym strukturell so verändert, dass das Substratspektrum der entstandenen Variante AAC6′-Ib-cr um Fluorchinolone wie Ciprofloxacin und Norfloxacin erweitert wird. Dies beruht auf der Neutralisierung der positiven Ladung an der 4′-NH_2_-Gruppe des Piperazinrings der genannten Fluorchinolone, wodurch deren Bindungsaffinität zu AAC6′-Ib-cr erhöht ist [28].

### Typ 3: Einschränkung des Zugangs des Antibiotikums zur Zielstruktur

Befindet sich die Zielstruktur eines Antibiotikums im Cytoplasma, muss das Molekül die Cytoplasmamembran passieren. Umfang und Geschwindigkeit dieses Durchtritts wird bestimmt durch verschiedene physikochemische Eigenschaften des jeweiligen Antibiotikums (z. B. Molekülgröße, 3D-Struktur, Oberflächenladung, Lipophilie, Affinität zu Membranstrukturen; [29]). Die darüberliegende Zellwand aus Peptidoglycan alleine (grampositive Zelle) ist für Antibiotika gut durchlässig, während die bei gramnegativen Zellen über der Zellwand befindliche zweite – äußere – Membran eine zusätzliche Barrierefunktion für hydrophile Moleküle, wie viele Antibiotika, hat. Dies bedingt primäre Resistenz von gramnegativen Bakterien gegen einige Antibiotikaklassen (Oxazolidinone, Lincosamide, Makrolide, Glycopeptide; [30]). Bei grampositiven Bakterien bewirkt das Fehlen einer äußeren Membran dagegen eine primäre Resistenz gegen Colistin, für dessen Wirkung die Desintegration der äußeren Membran essenziell ist [31].

In der äußeren Membran befinden sich zudem porenförmige, wassergefüllte Transmembrankanäle (Porine), durch die über passive Diffusion hydrophile Nährstoffe aufgenommen werden und die auch von Antibiotika mit den o. a. Eigenschaften (u. a. auch Chloramphenicol, Tetracyclin) genutzt werden. Anzahl und Typ der eingelagerten Porine sind genetisch reguliert und erlauben damit über eine Zugangskontrolle die Regulation der intrazellulären Antibiotikakonzentration [32].

Eine effizientere, da aktive Reduktion der intrazellulären Antibiotikakonzentration erfolgt durch in die Cytoplasmamembran eingelagerte Effluxpumpen. Unter Nutzung chemischer Energie in Form von ATP (ATP Binding Cassette, ABC-Typ-Pumpen) oder eines Protonengradienten über die Cytoplasmamembran (Effluxpumpen der Typen RND, MFS, SMR und MATE) exportieren diese Antibiotika aktiv aus dem Cytoplasma. Aufgrund ihrer geringen Substratspezifität werden sie als MDR-(Multidrug-resistance‑)Effluxpumpen bezeichnet. Mehrere solcher Pumpen mit unterschiedlichem Substratspektrum bestimmen beispielsweise bei *Pseudomonas aeruginosa* (s. oben) das Niveau der primären Resistenz gegen einige Antibiotika. Aufgrund der breiten, jedoch für das einzelne Antibiotikum geringen Substratspezifität ist die Exportleistung solcher Pumpen in der Regel gering.

Die nur bei gramnegativen Zellen vorkommenden RND-Typ-Effluxpumpen (**R**esistance, **N**odulation, Cell-**d**ivision) bestehen aus 3 Komponenten: Die Austrittsöffnung der Effluxpumpe an der Außenseite der Cytoplasmamembran wird über ein „Membranfusionsprotein“ (MFP) mit der Eintrittsöffnung eines Porins an der Innenseite der äußeren Membran verbunden. Durch den so vom Cytoplasma bis zum Extrazellularraum gebildeten Transportkanal werden Antibiotika durch beide Membranen ausgeschleust. Die bei Enterobakterien vorkommende AcrAB-TolC-Pumpe ist ein Beispiel. Die Expression der entsprechenden Gene *acrAB* (MFP, Effluxpumpe) und *tolC* (Porin) ist sowohl durch einen direkten Regulator (*acrR-*Genprodukt) als auch durch übergeordnete Regulatoren des *marRAB*-Operons positiv (MarA) bzw. negativ (MarR) reguliert. Über dieses Operon wird auch die Expression der unspezifischen Porine OmpC und OmpF synergistisch reguliert, indem die Reduktion dieser Porine mit der Erhöhung der Effluxpumpen gekoppelt erfolgt [33].

Tetracyclinresistenz beruht vor allem auf einem verringerten Zugang zur Zielstruktur, der Aminoacyl-tRNA-Bindungsstelle (A-Stelle) in der ribosomalen 50S-Untereinheit. Für eine klinisch relevante Resistenz gegen Tetracyclinderivate, jedoch nicht gegen Glycylcycline (z. B. Tigecyclin), sind bei gramnegativen und -positiven Bakterien Tetracyclin-spezifische Effluxpumpen des MFS-Typs (**M**ajor **F**acilitator **S**uperfamily) verantwortlich. Ein Beispiel ist TetA, das Tetracyclin im Antiport mit einem H^+^-Ion exportiert. Das entsprechende Gen *tetA* ist Plasmid-codiert [34].

Resistenz gegen Makrolide kann ebenfalls auf aktivem Austransport durch **M**akrolid-spezifische **Ef**fluxpumpen vom Typ MFS, wie Mef(A), beruhen [35].

Auch Chloramphenicol wird durch MDR-Effluxpumpen mit geringer Spezifität (z. B. AcrAB-TolC) aus der Zelle exportiert. Andere Effluxpumpen sind substratspezifischer: FloR bei gramnegativen sowie FexA und FexB bei grampositiven Bakterien vermitteln die Resistenz gegenüber Chloramphenicol und dessen Derivaten wie Florfenicol. Die Effluxpumpe Cml ist für Chloramphenicol spezifisch [36].

Eine weitergehende Übersicht über zugrunde liegende Mechanismen, klinische Bedeutung und Epidemiologie der wichtigsten Resistenzmechanismen findet sich im Onlinematerial.

## Ausblick auf Ansätze zur Entwicklung neuer Antibiotika und Alternativen

Die verschiedenen Varianten der 3 bakteriellen Basismechanismen für die Entwicklung von Antibiotikaresistenz (Typ 1, 2 und 3) sind ein Beispiel für die adaptiven Fähigkeiten von Bakterien, innerhalb kurzer Zeit auf Änderungen ihrer Umgebungsbedingungen erfolgreich zu reagieren. Die zunächst in den Anfängen der Antibiotikaentwicklung verfolgte Strategie, durch einfaches Screening von Naturstoffen neue Wirkstoffklassen zu identifizieren, zeitigte erste Erfolge mit der Entdeckung von Wirkstoffen, die an neuen Zielstrukturen angreifen und keine Parallelresistenz mit bekannten Antibiotika zeigen.

Neue molekularbiologische Methoden ermöglichten die Aufklärung der molekulargenetischen Grundlagen von Antibiotikaresistenzmechanismen. Daraus ergaben sich Ansätze, die antibiotische Wirksamkeit bekannter Wirkstoffe durch gezielte strukturelle Veränderungen zu erhöhen bzw. diese mit Inhibitoren für identifizierte resistenzvermittelnde Enzyme zu kombinieren [37]. Diese Strategien basierten allerdings auf der In-vitro-Analyse von molekularen Wechselwirkungen einer isolierten Zielstruktur mit einem Wirkstoff, auch unter Einsatz von 3D-Modelling-Techniken. Doch aufgrund der schnellen Anpassungsfähigkeit von Infektionserregern erwiesen sie sich nur als kurzfristige Option. So fanden sich bereits in präklinischen Studien häufig schon resistente Isolate. Der Wettlauf zwischen Bakterien und Mensch bei der Bekämpfung der Resistenzentwicklung gleicht dabei dem von „Hase und Igel“, da die Neuentwicklung eines effektiv wirksamen Antibiotikums neben dem Wirksamkeitsnachweis *in vitro* auch umfangreiche und langwierige klinische Prüfungen erfordert. Das verhindert einerseits eine rasche Markteinführung, was eine Aufrechterhaltung des bereits bestehenden Selektionsdrucks bedeutet. So können die Erreger sich durch inhärente genetische Mutations- und Rekombinationsmechanismen weiterentwickeln und Multiresistenzeigenschaften erwerben [37].

Erst Fortschritte in modernen molekulargenetischen Methoden, wie „whole genome sequencing“ „transcriptome profiling“ und „site-directed mutagenesis“ mittels CRISPR-cas, eröffneten den Zugang zur Aufklärung der Resistenzmechanismen auf zellulärer Ebene, die den In-vivo-Bedingungen deutlich näher kommen und zur Identifizierung weiterer, bislang nicht mit Antibiotikaresistenz assoziierter Mutationen führen. Teilweise betreffen diese Mechanismen Prozesse, die das mit dem Erwerb von Resistenz häufig einhergehende reduzierte „Fitnessniveau“ der Erreger kompensieren. Dazu zählen Stressantworten, wie sie durch bakterizid wirkende Antibiotika induziert werden, z. B. die Bildung reaktiver Sauerstoffspezies (ROS; [38]). Aus der Anwendung einer Bottom-up-Strategie ergäben sich neue Ansätze für das Design maßgeschneiderter Inhibitoren gegen entsprechende Zielstrukturen. Ein zusätzlicher Vorteil dabei wäre der kombinierte Angriff auf 2 unterschiedliche Zielstrukturen, wodurch die Wahrscheinlichkeit einer Resistenzentwicklung reduziert wird. Ob durch einen solchen Ansatz jedoch dauerhaft eine Resistenzentwicklung verhindert werden kann, ist nach den bisherigen Erfahrungen auch mit dem Einsatz rein chemisch-synthetisch hergestellter Antibiotika fragwürdig.

Mit Bakteriophagen (kurz Phagen) als anpassungsfähiger Alternative zu Antibiotika steht noch ein vielversprechender Ansatz zur Verfügung: Phagen infizieren bakterielle Wirtszellen, exprimieren ihre genetische Information unter Nutzung eigener und bakterieller Enzyme und setzen neu gebildete Phagenpartikel unter Zerstörung der Wirtszelle entweder direkt nach der Infektion (lytische Vermehrung) oder nach einer Phase latenter Existenz in der Wirtszelle (lysogene Vermehrung) frei. Eine besondere Eigenschaft von Phagen ist ihre Fähigkeit, sowohl die eigene als auch bakterielle DNA mit erhöhter Frequenz durch Mutationen bzw. Gentransfer (Transduktion, s. oben) zwischen Wirtszelle und Bakteriophagen zu verändern [39].

So begünstigt die ausgeprägte und durch Einsatz Phagen-eigener DNA-/RNA-Polymerasen ungenauere Replikation in der Wirtszelle die schnelle Anpassung einer Subpopulation mit Wirksamkeit gegen Phagen-resistente Wirtszellmutanten. Diese Subpopulation mutierter Phagen bildet den Ausgangspunkt für eine neue, wirksame Phagengeneration. Die Beschränkung der Phagenvermehrung auf die Anwesenheit von Wirtszellen und die schnelle Anpassungsfähigkeit auch unter Therapie spricht für einen breiten klinischen Einsatz von Phagen. Jedoch kann die genetische Variabilität von Phagen auch die Entwicklung von Antibiotikaresistenz bei Wirtszellen ermöglichen.

Angesichts der über hundert Millionen Jahre währenden Co-Existenz von Bakterien und Phagen ist nicht davon auszugehen, dass bakterielle Wirtszellen durch Phagen vollständig eliminiert werden, da damit zugleich die Grundlage für deren eigene Vermehrung entfallen würde. Dies spricht für den Einsatz kombinierter Therapiestrategien (Phagen und Antibiotika). Derzeit liegen allerdings noch zu wenige offizielle klinische Studiendaten vor, sondern vor allem Erfahrungen aus Einzelfallberichten [11].

## Fazit

Die Entwicklung von Antibiotikaresistenz ist eine evolutionär inhärente Eigenschaft von Bakterien. Hohe Wachstumsraten bei zugleich einfacher Chromosomenausstattung ermöglichen eine rasche Anpassung an Antibiotikastress durch Mutationen sowie den interzellulären Transfer von Resistenzgenen und tragen wesentlich zum zeitnahen Verlust therapeutischer Wirksamkeit bei. Eine Therapie mit Bakteriophagen stellt eine mögliche Option dar, die deren adaptive Eigenschaften nutzt, um bereits während der Therapie auf die Entwicklung bakterieller Resistenzen zu reagieren.

Die konsequente Beachtung wichtiger Grundregeln einer antibiotischen Therapie kann weiterhin dazu beitragen, den Prozess einer zunehmenden Resistenzentwicklung zumindest zu verlangsamen. Dazu gehören eine gezielte, diagnostisch abgesicherte Auswahl wirksamer Antibiotika, deren Einsatz in ausreichender Konzentration und über einen angemessenen, möglichst kurzen Zeitraum, optimale Hygienemaßnahmen im Umgang mit infizierten Patienten, insbesondere auf Intensivstationen, und ein Verbot des Antibiotikaeinsatzes zu nichttherapeutischen Zwecken [12].

## Supplementary Information

Tabelle: Ausgewählte Mechanismen bakterieller Antibiotikaresistenz

## Data Availability

Ein pdf-Dokument dieses Artikels ist online verfügbar auf den Seiten des Paul-Ehrlich-Instituts unter: https://www.pei.de/SharedDocs/Downloads/DE/institut/veroeffentlichungen-von-paul-ehrlich/1906-1914/1911-salvarsantherapie.pdf?__blob=publicationFile&v=2.
