# Supplementary material for: Molecular mechanisms underlying the development and spread of antibiotic resistance
Source: Bundesgesundheitsblatt Gesundheitsforschung Gesundheitsschutz. 2026 Apr 9;69(5):512–20. [Article in German] doi: 10.1007/s00103-026-04231-9 (PMC13132922; doi:10.1007/s00103-026-04231-9)
Supplement: Supplementary file 1 — Tabelle: Ausgewählte Mechanismen bakterieller Antibiotikaresistenz [file 103_2026_4231_MOESM1_ESM.pdf]

**Antibiotikum-klasse**	**Untergruppe**	**erfasstes Erregerspektrum**	**natürlich resistente Erreger**	**Erworbener Resistenzmechanismus**	**Resistenz typ[1)]**	**Klinische Resistenz[2)]**	**Zielstruktur**	**Lokalisation**
Sulfonamide	Sulfamethoxazol	*Streptococcus (S.) pneumoniae*, *Neisseria (N.) meningitidis*, Chlamydia Aktinomycetes, Nocardia,		Dihydropteroatsynthetase (DHPS)-Mutation	1	(+)	DHPS	Cytoplasma
Diamino-pyrimidine	Trimethoprim	die meisten aeroben Bakterien	Campylobacter *Tropheryma (T.) whippelii*	Dihydrofolatreductase (DHFR)-Mutation<br>DHFR-Überexpression	1<br><br>1	(+)<br><br>(+)	DHFR	Cytoplasma
Fluorchinolone	Ciprofloxacin, Levofloxacin, Norfloxacin	Gram-negative, Enterobacteriaceae, *Pseudomonas (P.) aeruginosa, Acinetobacter (A.) baumannii*	Enterococcus, Staphyloccus Streptococcus Topoisomerase (Topo) IV ist empfindliche Zielstruktur	*gyr*A (Gyrase A-)/<br>*par*C (Topo IV A-Mutation),<br>*gyr*B (Gyrase B-)/<br>*par*E (Topo IV B-Mutation)<br>QnrA,<br>AcrAB-TolC multiple drug-resistance (MDR)-Efflux,<br>OmpF Porinreduktion,<br>QepA (spezifischer Efflux)<br>AAC(6')-Ib-cr	1<br><br>1<br><br>1<br>3<br><br>3<br>3<br>2	(+)<br><br>(-)<br><br>(-)<br>(-)<br><br>(-)<br>(-)<br>(-)	Gyrase-DNA-Komplex,<br>Topo IV-DNA Komplex,<br><br><br>(Norfloxacin u. Ciprofloxacin)	Cytoplasma
	Moxifloxacin, Trovafloxacin, Clinafloxacin	*Streptococcus (S.) pyogenes*; *S.pneumoniae*, Staphylococcus, (Topo IV empfindliche Zielstruktur) Haemophilus, Legionella, Chlamydia, Mycoplasma	aerobe Gram-negative, Pseudomonas, Acinetobacter (Gyrase ist empfindliche Zielstruktur)	*parC*-/*gyr*A-<br>*par*E/*gyr*B	1<br>1	(+)<br>(+/-)	Topo IV-DNA-Komplex, Gyrase- DNA-Komplex	Cytoplasma
Oxazolidinone	Linezolid	Enterococcus, Streptococcus Staphylococcus Clostridioides	Gram-negative, (MDR-Efflux)	23S rRNA Mutation,<br>50S Ribosom L3-, L4-Mutation<br>23S rRNA-Methylierung (cfr)	1<br>1<br>1	(+)<br>(+)<br>(+/-)	Ribosom, 50S-Untereinheit, Peptidyltrans-ferase Center	Cytoplasma

[1)] : Resistenztyp 1: Veränderung der Zielstruktur; Resistenztyp 2: Inaktivierung des Antibiotikums; Resistenztyp 3: Reduzierter Zugang zur Zielstruktur.

[2)] : (+): klinische Resistenz durch den Resistenzmechanismus - auch einzeln; (-): keine klinische Resistenz durch Resistenzmechanismus einzeln;
(-) kombi: klinische Resistenz durch Resistenzmechanismus nur möglich in Kombination mit anderem - z.B. unspezifischem - Mechanismus;
(+/-): klinische Resistenz möglich abhängig von der jeweiligen Mutation.

**Antibiotikum-klasse**	**Untergruppe**	**erfasstes Erregerspektrum**	**natürlich resistente Erreger**	**Erworbener Resistenzmechanismus**	**Resistenz-typ**	**Klinische Resistenz**	**Zielstruktur**	**Lokalisation**
Aminoglycosid-<br>Aminocyclitole<br>("Amino-<br>glycoside")	Gentamicin,<br>Amikacin,<br>Tobramycin,<br>Kanamycin,<br>Sisomicin,<br>Isepamicin	Enterobacteriaceae<br>*P.aeruginosa,*<br>*A.baumannii*<br>Staphylococcus,<br>Enterococcus<br>(variabel)	Enterococcus,<br>(z.T. hochgradig)<br>Streptococcus,<br>Anaerobier<br>(Clostridioides,<br>Bacterioides)<br>Chlamydia	Aminoglycosid-modifizierende<br>Enzyme:<br>Nucleotidyltransferase (ANT),<br>Phosphotransferease (APH),<br>Acetyltransferase (AAC)<br>(jeweils Species-spezifisches<br>Erregerspektrum)<br>16S rRNA-Methylierung<br>(Arm, Rmt)<br>(Npm)	2<br><br><br><br><br><br><br>1	(+)<br><br><br><br><br><br><br>(+)	Ribosom 70S,<br>Stabilisierung<br>Interaktion<br>30S mit 50S,<br>Misstranslation<br>Bildung defekter<br>Proteine)<br><br>16S-rRNA isoliert<br>16S rRNA in 30S	Cytoplasma
Amphenicole	Chloramphe-<br>nicol	Gram-negative inkl.<br>Salmonella Typhi<br>Salmonella non-Typhi<br>sporenlose Anaerobier<br>Burkholderia	*P.aeruginosa*,<br>Mycobacteria-	23S rRNA-Mutation<br>Chloramphenicol-<br>acetyltransferase (CAT)<br>unspez. Efflux (AcrAB-TolC)<br>spezifischer Efflux (ClmBr)	1<br>2<br><br>3<br>3	(-)<br>(+)<br><br>(-)<br>(+)	Ribosom, 50S<br>Untereinheit, 23S<br>rRNA,<br>A-Stelle mit<br>Aa-tRNA	Cytoplasma
Macrolide	Erythromycin<br>Clarithromycin	Gram-positive	Gram-negative	23S rRNA Methylierung (Erm)<br>induzierbar/konstitutiv)<br>23S rRNA Mutation (*rpl*D,*rpl*V)<br>Makrolid-Esterasen (Ere)<br>Spezifischer Efflux (Mef, Msr)	1<br><br>1<br>2<br>3	(+)<br><br>(+)<br>(-)<br>(+)	Ribosom,<br>50S-Untereinheit<br>23S rRNA und<br>L4, L22 Proteine.	Cytoplasma
Tetracycline	Tetracyclin	aerobe Gram-positive<br>(Staphylococcus,<br>Streptococcus,<br>*S. pneumoniae*,<br>Propionibacteria)<br>Gram-negative<br>*(Haemophilus (H.)*<br>*influenzae*,<br>Enterobacteriaceae,<br>Borrelia, Moraxella,<br>Vibrio, Francisella,<br>Mycoplasma)	*P.aeruginosa*,<br>Proteus	Mutation 16S rRNA (A-Stelle)<br>Mutation 30S *rps*J (S10)<br>ribosomales Schutzprotein<br>(TetM, TetO, TetS, TetW)<br>enzymatische Inaktivierung<br>(TetX Oxidase)<br>reduzierte Porin Expression<br>(OmpC, OmpF)<br>Tetracyclin-spezifischer Efflux<br>(TetA - TetE) induzierbar<br>unspez. Efflux (AcrAB-TolC),<br>induzierbar	1<br>1<br>1<br><br>2<br><br>3<br><br>3<br><br>3	(+)<br>(-)<br>(+)<br><br>(-)<br><br>(-)<br><br>(+)<br><br>(-)	Ribosom,<br>A-Stelle,<br>30S rpsJ<br>16S rRNA und<br>23S rRNA in<br>70S Ribosom	Cytoplasma

[1] : Resistenztyp 1: Veränderung der Zielstruktur; Resistenztyp 2: Inaktivierung des Antibiotikums; Resistenztyp 3: Reduzierter Zugang zur Zielstruktur.

[2] : (+): klinische Resistenz durch den Resistenzmechanismus - auch einzeln; (-): keine klinische Resistenz durch Resistenzmechanismus einzeln;
(-) kombi: klinische Resistenz durch Resistenzmechanismus nur möglich in Kombination mit anderem - z.B. unspezifischem - Mechanismus;
(+/-): klinische Resistenz möglich abhängig von der jeweiligen Mutation.

**Antibiotikum-klasse**	**Untergruppe**	**erfasstes Erregerspektrum**	**natürlich resistente Erreger**	**Erworbener Resistenzmechanismus**	**Resistenz-typ**	**Klinische Resistenz**	**Zielstruktur**	**Lokalisation**
Glycylcycline	Tigecyclin	*S.aureus* inkl. (MRSA), Glycopeptid-intermediär *S.aureus* (GISA)<br>*S.pneumoniae, inkl.* Penicillin-resistente *S.pneumoniae*(PRSP)<br>Enterococcus (VRE)<br>Enterobacteriaceae<br>*A.baumannii,*<br>Stenotrophomonas<br>Clostridioides,<br>Peptostreptococcus<br>Chlamydia,<br>Mycoplasma	Proteus,<br>Serratia,<br>Morganella,<br>*P.aeruginosa* (unspezifischer Efflux)	Ribosomales Schutzprotein (TetM)<br>Enzymatische Inaktivierung (TetX) Oxidase<br>Tet-spezifischer Efflux (TetA, modifiziert)<br>unspezifischer MDR Efflux (Überexpression)	1<br><br>2<br><br>3<br><br>3	(+) >1µg(ml<br>(+) kombi<br><br>(+) >1µg/ml<br>(+/-)	Ribosom30 S,<br>Helix H34	Cytoplasma
Streptogramine	Dalfopristin (Dalfo) (Streptogramin A-Typ)<br>Quinupristin (Quinu) (Streptogramin B-Typ)	Streptococcus,<br>*S.pneumoniae* (PRSP),<br>*S.aureus* (MRSA)<br>Legionella,<br>Mycoplasma,<br>Clostridioides,<br>Chlamydia	Bacteroides,<br>Gram-negative aerobe Stäbchen	O-Acetyltransferase (VanD)<br><br>23S rRNA Methylase (Erm)<br><br>Esterhydrolase, Lyase (Vgb)<br><br>Efflux (MsrC)	1<br><br>1<br><br>2<br><br>3	(+) kombi + Dalfo+ Quinu<br>(+) kombi +Dalfo<br>(-) Quinu<br>(+) kombi +Dalfo	Ribosom,<br>Peptidyltrans-ferase-Center (PTC)<br>Dalfo (50S),<br>23S rRNA (U2506-G2583)<br>Quinu (50S)<br>23S rRNA (A2062)	Cytoplasma

[1] : Resistenztyp 1: Veränderung der Zielstruktur; Resistenztyp 2: Inaktivierung des Antibiotikums; Resistenztyp 3: Reduzierter Zugang zur Zielstruktur.

[2] : (+): klinische Resistenz durch den Resistenzmechanismus - auch einzeln; (-): keine klinische Resistenz durch Resistenzmechanismus einzeln;
(-) kombi: klinische Resistenz durch Resistenzmechanismus nur möglich in Kombination mit anderem - z.B. unspezifischem - Mechanismus;
(+/-): klinische Resistenz möglich abhängig von der jeweiligen Mutation.

**Antibiotikum-klasse**	**Untergruppe**	**erfasstes Erregerspektrum**	**natürlich resistente Erreger**	**Erworbener Resistenzmechanismus**	**Resistenz-typ**	**Klinische Resistenz**	**Zielstruktur**	**Lokalisation**
β-Lactame	Benzylpenicillin (Penicillin G)	Gram-positive Streptococcus, *S. aureus* (*bla*Z$^-$) *S.pneumonioae,* Bacillus, Neisseria, Corynebacteria, Treponema, Pasteurella, Gram-negative Anaerobier	Gram-negative, Enterobacteria inkl. Salmonella *S. aureus* (*bla*Z$^+$) Enterococcus	β-Lactamase (Bla)<br>Penicillinase (BlaZ)<br><br>erhöhter MDR-Efflux<br>reduzierter Influx (Porin)	2<br><br><br>3<br>3	(+)<br><br><br>(-) kombi<br>(-) kombi	Zellwand, Peptidoglycan, Transpeptidase (Penicillin-Binde-protein, PBP)	Zellwand, Murein-schicht
	Aminopenicillin (Amoxicillin)	Gram-positive Streptococcus, Enterococcus Corynebacteria, Neisseria, Treponema, *E.coli*, Salmonella (teilw.), Shigella	*P.aeruginosa,* Enterobacteria Citrobacter, Enterobacter), Klebsiella *Enterococcus (E.) faecium*	mutiertes PBP<br>Penicillinase (BlaZ)<br>Bla (A): Bla(ESBL)<br>Bla (C): BlaAmpC (konstitutiv)<br>erhöhter MDR Efflux	1<br>2<br><br>2<br>3	(+)<br>(+)<br><br>(+)<br>(-)	Zellwand, Peptidoglycan, Transpeptidase (PBP)	Zellwand, Murein-schicht
	Isoxazolyl-penicillin (Flucloxacillin)	*S.aureus* (*bla*Z$^+$), (*S.pneumoniae*), (Streptococcus)	Gram-negative	neues mutiertes PBP PBP2a (Expression induzierbar/konstitutiv)	1	(+)	Zellwand, Peptidoglycan, Transpeptidase (PBP)	Zellwand, Murein-schicht
	Acylaminopeni-cillin (Piperacillin)	Ähnlich wie Aminopenicilline, *P.aeruginosas, H.influenzae* , Anaerobier (*Bacteroides fragilis*)	*S.aureus* (*bla*Z$^+$) Enterobacteria (Citrobacter, Enterobacter), Klebsiella	BlaZ (Penicillinase)<br><br>Bla (C): BlaAmpC (konstitutiv)	2<br><br>2	(+)<br><br>(+)	Zellwand, Peptidoglycan, Transpeptidase (PBP)	Zellwand, Murein-schicht

1) : Resistenztyp 1: Veränderung der Zielstruktur; Resistenztyp 2: Inaktivierung des Antibiotikums; Resistenztyp 3: Reduzierter Zugang zur Zielstruktur.

2) : (+): klinische Resistenz durch den Resistenzmechanismus - auch einzeln; (-): keine klinische Resistenz durch Resistenzmechanismus einzeln;
(-) kombi: klinische Resistenz durch Resistenzmechanismus nur möglich in Kombination mit anderem - z.B. unspezifischem - Mechanismus;
(+/-): klinische Resistenz möglich abhängig von der jeweiligen Mutation.

Antibiotikum-klasse	Untergruppe	erfasstes Erregerspektrum	natürlich resistente Erreger	Erworbener Resistenzmechanismus	Resistenz-typ	Klinische Resistenz	Zielstruktur	Lokalisation
β-Lactame (Forts.)	Cephalosporine (1. Generation) Cefazolin	Staphylococcus, *S. aureus* (*blaZ*$^{+}$) Gram-negative (*E. coli, K. pneumoniae, P. mirabilis*)	Enterococcus, *S.pneumoniae*, Enterobacteria (AmpC^{+}), *A.baumannii*, Mycoplasma, *Bacteroides* (*B.*) *fragilis*, *H.influenzae*	neues mutiertes PBP PBP2a, PBP3<br>Bla (C): BlaAmpC (konstitutiv)	1<br><br>2	(+)<br><br>(+)	Zellwand, Peptidoglycan, Transpeptidase (PBP)	Zellwand, Murein-schicht
	Cephalosporine (2. Generation) Cefuroxim	*H.influenzae* (Bla-/+) *E.coli* (BlaTEM-1-/+) Neisseria (Bla-/+) Streptococcus, *S.aureus*	Morganella, Serratia, Proteus, *P.aeruginosa*, Enterococcus, Mycoplasma, Chlamydia	neues mutiertes PBP2a β-<br><br>Bla (A): BlaTEM (ESBL)	1<br><br>2	(+)<br><br>(+)	Zellwand, Peptidoglycan, Transpeptidase (PBP)	Zellwand, Murein-schicht
	Cephalosporine (3a. Generation) Oxyimino-Cephalosporine Cefotaxim	Enterobacteriaceae (speziell *E.coli, K. pneumoniae, P. mirabilis*, *H.influenzae* (Bla$^{-/+}$)	*B.fragilis* Enterococcus, Mycoplasma, Chlamydia, Listeria	neues PBP2a<br>Bla (A): Bla (ESBL)<br>Bla (C): BlaAmpC (konstitutiv)<br>-	1<br><br>2<br>2	(+)<br><br>(+)<br>(+)	Zellwand, Peptidoglycan, Transpeptidase (PBP)	Zellwand, Murein-schicht
	Cephalosporine (3b. Generation) Ceftazidim	Enterobacteriaceae (speziell *E.coli*) Klebsiella, Proteus, *H.influenzae* (Bla-/+) *P.aeruginosa*	*B.fragilis* Enterococcus, Mycoplasma, Chlamydia, Listeria, Campylobacter, Clostridioides	neues PBP2a<br>Bla (A): (ESBL)<br>Bla (C) BlaAmpC (konstitutiv)<br>Enterobacter, Citrobacter<br>erhöhter, unspezifischer MDR-Efflux<br>Porin OprD Verlust	1<br>2<br>2<br><br>3<br><br>3	(+)<br>(+)<br>(+)<br><br>(-)<br><br>(-)	Zellwand, Peptidoglycan, Transpeptidase (PBP)	Zellwand, Murein-schicht

[1)] : Resistenztyp 1: Veränderung der Zielstruktur; Resistenztyp 2: Inaktivierung des Antibiotikums; Resistenztyp 3: Reduzierter Zugang zur Zielstruktur.

[2)] : (+): klinische Resistenz durch den Resistenzmechanismus - auch einzeln; (-): keine klinische Resistenz durch Resistenzmechanismus einzeln;
(-) kombi: klinische Resistenz durch Resistenzmechanismus nur möglich in Kombination mit anderem - z.B. unspezifischem - Mechanismus;
(+/-): klinische Resistenz möglich abhängig von der jeweiligen Mutation.

**Antibiotikum-klasse**	**Untergruppe**	**erfasstes Erregerspektrum**	**natürlich resistente Erreger**	**Erworbener Resistenzmechanismus**	**Resistenz-typ**	**Klinische Resistenz**	**Zielstruktur**	**Lokalisation**
β-Lactame (Forts.)	Cephalosporine Siderophor-C. Cefiderocol	aerobe Gram-negative, Enterobacteriaceae *P.aeruginosa, A.baumannii,* Stenotrophomonas, Burkholderia (auch Amikacin^{R}, Cefipim^{R}, Fluorchinolon^{R}) auch β-Lactamase^{+} (Bla (A), (B), (C), (D))	aerobe Gram-positive, Anaerobe	mutiertes PBP<br>β-Lactamase -Kombi<br>Bla (B): Bla-Metallo (NDM-5)<br>Bla (C): BlaAmpC<br>mutiertes Eisentransportsystem (Siderophor-Biosynthese)	(1)<br><br>(2)<br><br><br>(3)	(+)<br><br>(+)<br><br><br>(+)	Zellwand, Peptidoglycan, Transpeptidase (PBP)	Zellwand, Murein-schicht
	Carbapeneme Imipenem	*A.baumannii, P.aeruginosa,* Enterobacteriaceae (ESBL -/+)	Stenotropho-monas, *S.aureus* (MRSA) Enterococcus (Ampicillin^{R}),	Bla (Carbapenemase)<br>Bla (A): BlaKPN<br>Bla (B): Bla-Metallo<br>Bla (D): BlaOXA<br>reduzierte Porin-Expression<br>MDR Effluxpumpen Überexpression<br>mutiertes PBP	2<br>2<br>2<br>2<br>3<br>3<br><br>1	(+)<br>(+)<br>(+)<br>(+)<br>(+)<br>(-)<br><br>(-)	Zellwand, Peptidoglycan, Transpeptidase (PBP)	Zellwand, Murein-schicht
	Monobactame Azthreonam	Gram-negative aerobe Stäbchen *P.aeruginosa*, Aeromonas (hohe PBP3 Affinität)	*A.baumannii;* Stenotropho-monas, Burkholderia, Bacteroides, Gram-positive Anaerobier	Bla (A): BlaESBL; (TEM, SHV, CTX-M)<br>Bla (C): BlaAmpC<br>Bla (D): BlaOXA	2<br><br>2<br>2	(+)<br><br>(+)<br>(+)	Zellwand, Peptidoglycan, Transpeptidase (PBP)	Zellwand, Murein-schicht

[1)] : Resistenztyp 1: Veränderung der Zielstruktur; Resistenztyp 2: Inaktivierung des Antibiotikums; Resistenztyp 3: Reduzierter Zugang zur Zielstruktur.

[2)] : (+): klinische Resistenz durch den Resistenzmechanismus - auch einzeln; (-): keine klinische Resistenz durch Resistenzmechanismus einzeln;
(-) kombi: klinische Resistenz durch Resistenzmechanismus nur möglich in Kombination mit anderem - z.B. unspezifischem - Mechanismus;
(+/-): klinische Resistenz möglich abhängig von der jeweiligen Mutation.

**Antibiotikum-klasse**	**Untergruppe**	**Erregerspektrum**	**Natürliche Resistenz**	**Erworbene Resistenz**	**Resistenz-typ**	**Klinische Resistenz**	**Zielstruktur**	**Lokalisa-tion**
Glycopeptide	Vancomycin	Enterococcus,<br>*S.aureus* (MRSA)<br>Streptococcus<br>*S.pneumoniae* (PRSP)<br>Clostridioides,<br>Corynebacterium<br>Gram-positive Anerobier	Gram-negative	Zellwand-Umbau<br>VanA - VanN;<br>(D-Alanyl-D-Lactat-Ligase)<br>Überexpression von (PBP) -<br>verstärkte Bildung<br>von (D-Alanyl-D-Alanin)	1<br><br><br>1<br>1	(+)<br><br><br>(+)<br>(+)	Peptidoglycan,<br>Muropeptid,<br>D-Alanyl-D-Alanin-Dipeptid	Zellwand,<br>Murein-schicht
Lipopeptide	Daptomycin	Gram-positive<br>*S.aureus*<br>(MRSA, VISA,VRSA)<br>Enterococcus<br>(GRE)	Gram-negative (impermeable LPS-Schicht der Äußeren Membran)	Lysylphosphatidylglycerol-synthese (MprF)<br>RNA-Polymerase-Untereinheiten RpoBC	1<br><br>(1)	(+)<br><br>(-)	Phosphatidyl-glycerol	Cytoplasma membran
Polymyxine	Colistin	Gram-negative	Gram-positive	Phosphoethanolamin-transferase (Mcr-1)<br>Reduzierte/unterbrochene LPS-Synthese (LpxABC)<br>Aktivierung der 4-Amino-L-arabinose-Transferase (PmrABCD<br>Glycosylierung von LPS (CcrABC)	1<br><br>1<br><br><br>1<br><br><br>1	(+)<br><br>(+)<br><br><br>(+)<br><br><br>(+)	Äußere Membran,<br>Lipid A	Äußere Membran

[1)] : Resistenztyp 1: Veränderung der Zielstruktur; Resistenztyp 2: Inaktivierung des Antibiotikums; Resistenztyp 3: Reduzierter Zugang zur Zielstruktur.

[2)] : (+): klinische Resistenz durch den Resistenzmechanismus - auch einzeln; (-): keine klinische Resistenz durch Resistenzmechanismus einzeln;
(-) kombi: klinische Resistenz durch Resistenzmechanismus nur möglich in Kombination mit anderem - z.B. unspezifischem - Mechanismus;
(+/-): klinische Resistenz möglich abhängig von der jeweiligen Mutation.
